# To implant or not to implant? That is the unsolved question concerning heart failure patients with non-LBBB

**DOI:** 10.1007/s12471-015-0778-2

**Published:** 2015-12-08

**Authors:** M. Zarse, B. Lemke

**Affiliations:** Klinikum Lüdenscheid, Medizinische Klinik III Kardiologie und Angiologie, Pauwelstr. 14, 58515 Lüdenscheid, Germany

With their article published in this issue of the Journal, Stipdonk and colleagues touch on a subject bearing significant clinical implications [1].

The inception of the concept of electrical cardiac resynchronisation therapy (CRT) was followed by an unprecedented clinical success story for heart failure patients suffering from bundle branch block. Initially we embraced this concept for all patients with long QRS duration independent of the morphology of the bundle branch block [2, 3]. As in all therapies, there were some non-responders; however, for a highly invasive therapy this was unacceptably high at 30–40 %. Therefore considerable efforts have been put into echocardiography to improve patient selection as well as optimise atrioventricular and interventricular stimulation patterns.

These echocardiographic studies revealed that also patients with smaller QRS durations might suffer from delayed left ventricular wall activation leading to another multicentre study feeding the aspiration to further expand the indication for CRT. However, the higher the rise, the greater the fall. Consecutive studies painstakingly showed that CRT therapy might not only be ineffective but actually detrimental for the patients [4].

In the present guidelines, echocardiographic parameters for left ventricular activation delay are irrelevant for the CRT indication. Meta-analysis of the landmark CRT studies displays a dependency of the responder rate on QRS duration and left bundle branch block (LBBB) morphology. Therefore, only LBBB morphology represents a class I indication for CRT, whereas non-LBBB morphology was downgraded to a class IIa (QRS > 150 ms) and IIb indication (QRS 120–150 ms), respectively [5].

This re-evaluation was backed up by the results of the long-term follow-up of the MADIT-CRT study which revealed an improved prognosis solely for patients with LBBB, whereas the prognosis for patients with non-LBBB was worsened [6]. Non-LBBB is associated with a conglomeration of diverse causes which might also show delay in left ventricular wall activation.

This dilemma is obvious: On the one hand there are many heart failure patients with non-LBBB who are at the end of the road for medical therapy and urgently need further treatment options. On the other hand, we need some certainty that we do not harm these patients by implanting a CRT-D system.

The guidelines concerning implantation of CRT-D in patients with non-LBBB are reflective of this dilemma. They advise us to implant these patients only after careful selection. However, they do not tell us how to select [4].

This represents the value of the article from the Maastricht Group. By demonstrating the feasibility of coronary venous electroanatomical mapping, they break new ground in proposing an individualised method of screening patients with non-LBBB for eligibility for CRT implantation, which might one day be part of the selection process.

In their study they draw the conclusion that neither QRS duration nor morphology are adequate discriminators for the selection process, already hinting that this selection might not be an easy one.

Likewise they consistently demonstrate that the latest activation region was consistently at the basal lateral wall, which raises a couple of questions which need to be answered in further systematic studies:

1. Do we really have to target our stimulation at the latest point of activation? This question is so important as it influences the way we interpret an electrical mapping which often displays a rather homogenous spread of activation with the last 10 or 20 % of the map being activated very slowly. This is also closely related to the question how epicardial and endocardial mapping correlate, which seems close according to our own experience. Basal stimulation at a point where the coronary venous anatomy is still rather broad would be challenging and implies a call for the more frequent use of quadripolar or mechanically fixable electrodes which, due to their special pre-specified form, would allow easier positioning of an electrode in this target region. Therefore, the question has to be answered as to whether stimulating at this point yields haemodynamic benefit or if it is not sufficient to target some point where 80 or 90 % homogenous activation has passed and is located less basally. This brings up the next question:
2. Does our electroanatomical activation correlate to our haemodynamic activation? There are some promising studies showing a correlation when comparing electrode delays, but further substantiating the above study is a sine qua non for further use of this method and it brings up the final and all-dominant question:
3. Will heart failure patients with non-LBBB selected for and implanted according to individualised electroanatomical mapping show clinical benefit in the furnace of a randomised clinical trial?

There is a lot of a fascinating and exciting but also sobering and disillusioning road ahead to be filled with studies like the above, to form the pieces of a puzzle which some day might yield the whole picture, allowing us to finally answer the question if and how to implant these patients.

## Funding

None.

## Conflict of interests

None declared.
